# Phenotypic and Genotypic Adaptations in Pseudomonas aeruginosa Biofilms following Long-Term Exposure to an Alginate Oligomer Therapy

**DOI:** 10.1128/mSphere.01216-20

**Published:** 2021-01-20

**Authors:** Juliette L. Oakley, Rebecca Weiser, Lydia C. Powell, Julian Forton, Eshwar Mahenthiralingam, Philip D. Rye, Katja E. Hill, David W. Thomas, Manon F. Pritchard

**Affiliations:** a Advanced Therapies Group, Oral and Biomedical Sciences, School of Dentistry, College of Biomedical and Life Sciences, Cardiff University, Cardiff, United Kingdom; b Cardiff School of Bioscience, Cardiff University, Cardiff, United Kingdom; c Department of Paediatric Respiratory Medicine/Cystic Fibrosis Unit, Children’s Hospital for Wales, Cardiff, United Kingdom; d AlgiPharma AS, Sandvika, Norway; Antimicrobial Development Specialists, LLC

**Keywords:** cystic fibrosis, *Pseudomonas aeruginosa*, alginate oligosaccharide, biofilm, bead model

## Abstract

Chronic Pseudomonas aeruginosa lung infections in cystic fibrosis (CF) evolve to generate environmentally adapted biofilm communities, leading to increased patient morbidity and mortality. OligoG CF-5/20, a low-molecular-weight inhaled alginate oligomer therapy, is currently in phase IIb/III clinical trials in CF patients. Experimental evolution of P. aeruginosa in response to OligoG CF-5/20 was assessed using a bead biofilm model allowing continuous passage (45 days; ∼245 generations). Mutants isolated after OligoG CF-5/20 treatment typically had a reduced biofilm-forming ability and altered motility profile. Genotypically, OligoG CF-5/20 provided no selective pressure on genomic mutations within morphotypes. Chronic exposure to azithromycin, a commonly prescribed antibiotic in CF patients, with or without OligoG CF-5/20 in the biofilm evolution model also had no effect on rates of resistance acquisition. Interestingly, however, cross-resistance to other antibiotics (e.g., aztreonam) was reduced in the presence of OligoG CF-5/20. Collectively, these findings show no apparent adverse effects from long-term exposure to OligoG CF-5/20, instead resulting in both fewer colonies with multidrug resistance (MDR)-associated phenotypes and improved antibiotic susceptibility of P. aeruginosa.

**IMPORTANCE** The emergence of multidrug-resistant (MDR) pathogens within biofilms in the cystic fibrosis lung results in increased morbidity. An inhalation therapy derived from alginate, OligoG CF-5/20, is currently in clinical trials for cystic fibrosis patients. OligoG CF-5/20 has been shown to alter sputum viscoelasticity, disrupt mucin polymer networks, and disrupt MDR pseudomonal biofilms. Long-term exposure to inhaled therapeutics may induce selective evolutionary pressures on bacteria within the lung biofilm. Here, a bead biofilm model with repeated exposure of P. aeruginosa to OligoG CF-5/20 (alone and in combination with azithromycin) was conducted to study these long-term effects and characterize the phenotypic and genotypic adaptations which result. These findings, over 6 weeks, show that long-term use of OligoG CF-5/20 does not lead to extensive mutational changes and may potentially decrease the pathogenicity of the bacterial biofilm and improve the susceptibility of P. aeruginosa to other classes of antibiotics.

## Abstract (precis)

The emergence of multidrug-resistant (MDR) pathogens within biofilms in the cystic fibrosis lung results in increased morbidity. An inhalation therapy derived from alginate, OligoG CF-5/20, is currently in clinical trials for cystic fibrosis patients.

## INTRODUCTION

Cystic fibrosis (CF) is an autosomal recessive disorder, affecting up to 1 in 2,500 live births (1), with higher incidence in the United States, Europe, and Australia (2). The disease is characterized by mutations in the CF transmembrane conductance regulator (CFTR) gene, resulting in impaired functioning of epithelial chloride channels (3) and a biochemically altered viscous mucus (4). CF is associated with recurrent lung infection and chronic inflammation, which ultimately lead to impaired lung function and premature death (5). Prolonged antibiotic treatment has played a vital role in controlling CF infections and contributed to the increasing median life expectancy of CF patients (6). Chronic reliance on antibiotics by CF patients, however, produces considerable selective pressures on the lung biofilm community. The opportunistic pathogen Pseudomonas aeruginosa typically colonizes the lungs of CF patients at 8.8 years old (7), where it then undergoes considerable selective pressure from systemic and chronically inhaled antibiotics, the host immune system, and oxidative stress within the CF lung biofilm (8). The inability of the immune response or long-term, high-dose, antimicrobial treatments to totally eradicate P. aeruginosa is witness to the fitness advantage of this evolutionary process (9).

The environmental stress stimuli in the CF lung are reflected in the extensive phenotypic and genotypic adaptations observed in CF P. aeruginosa populations (10). With chronic colonization, later-stage bacterial isolates are distinctly different from pioneer colonizers, displaying well-defined characteristics such as loss of motility, mucoidy, reduced growth rates, increased antibiotic resistance, and defective quorum sensing (QS) signaling (8). Furthermore, mutations in P. aeruginosa to the alginate-producing mucoid phenotype or highly adherent small colony variants (SCVs) occur with increased resistance to host defenses (11). Eventually, *de novo* mutations lead to evolution of antibiotic resistance in individual patients (12, 13), enabling the CF lung to become colonized by multidrug-resistant (MDR) pathogens, particularly P. aeruginosa (10). Of those patients positive for P. aeruginosa in 2018, 16.9% were reported by the Cystic Fibrosis Foundation to carry MDR strains (https://www.cff.org/Research/Researcher-Resources/Patient-Registry/2018-Patient-Registry-Annual-Data-Report.pdf).

A new CF inhalation therapy, OligoG CF-5/20, is currently in phase IIb/III clinical trials in CF patients (www.ClinicalTrials.gov; identifiers NCT03698448 and NCT03822455). OligoG CF-5/20 is a low-molecular-weight polydisperse alginate oligosaccharide (number average molecular weight [Mn], 3,200) shown to be well tolerated with no toxic/adverse events in randomized, placebo-controlled, dose-escalation clinical trials. Lung scintigraphic studies demonstrated a 38.6% deposition of the dry powder inhalation (14). *Ex vivo* studies have also demonstrated the ability of OligoG CF-5/20 to alter the viscoelastic properties and disrupt the complex mucin polymer networks of CF sputum (14). Furthermore, OligoG CF-5/20 has been shown to possess antibiofilm properties (15, 16) and enhance antibiotic activity (16, 17) against P. aeruginosa. Effects are mediated via extracellular polymeric substance (EPS) disruption (15), inhibition of QS signaling (18), and alteration of bacterial surface charge and motility (17, 19).

While antimicrobial susceptibility testing is predominantly based on laboratory culture of planktonically growing bacteria, it has become increasingly clear that localized chronic infections are principally the result of intractable bacterial biofilms (20). With antimicrobial resistance in biofilms being up to 1,000-fold greater than that of planktonically growing isogenic strains (21), biofilm models have been increasingly employed to examine bacterial evolutionary pathways. Within CF, biofilm models incorporate many of the characteristics of a CF lung ecosystem, with slow growth in an entangled polymeric network, facilitating horizontal gene transfer between MDR bacteria, which can withstand repeated antimicrobial dosing (22). A number of biofilm evolutionary models have been reported in the literature (23) with the complex structure of the biofilm environment demonstrating increased ecological diversity and productivity (24). In the present study, a bead biofilm model was used that incorporated regular cycles of surface colonization, biofilm assembly, and dispersal. Importantly, *in vitro* long-term evolution experiments such as bead biofilm models have been shown to mimic P. aeruginosa adaptation in the CF lung (10, 25, 26).

We have previously shown that planktonic subculture of P. aeruginosa (PAO1) for 21 days in escalating concentrations of OligoG CF-5/20 did not result in loss of antimicrobial activity (17). The present study aimed to determine the effects of prolonged exposure of P. aeruginosa biofilms to OligoG CF-5/20 with regard to the evolved genotypic and phenotypic characteristics of different colony morphotypes to determine impact on bacterial evolution. This study also determined the acquisition of resistance to azithromycin (AZM) (an antibiotic commonly used in the treatment of CF) in the presence and absence of sublethal concentrations of OligoG CF-5/20 (27). Positive impacts of selective pressure on biofilm growth (negative impact for the host) by OligoG CF-5/20 might be expected to manifest themselves by increased development of SCVs and mucoid phenotypes, both of which are associated with increased resistance to antimicrobial therapy. This study, however, demonstrates that OligoG CF-5/20 exposure did not cause extensive mutational changes in P. aeruginosa but instead appeared to reduce both MDR-associated phenotypes and prevalence of antibiotic-resistant phenotypes. The mechanisms underpinning this are explored.

## RESULTS

### Prolonged exposure of P. aeruginosa to OligoG CF-5/20 results in decreased morphotype diversity.

**FIG 1:**
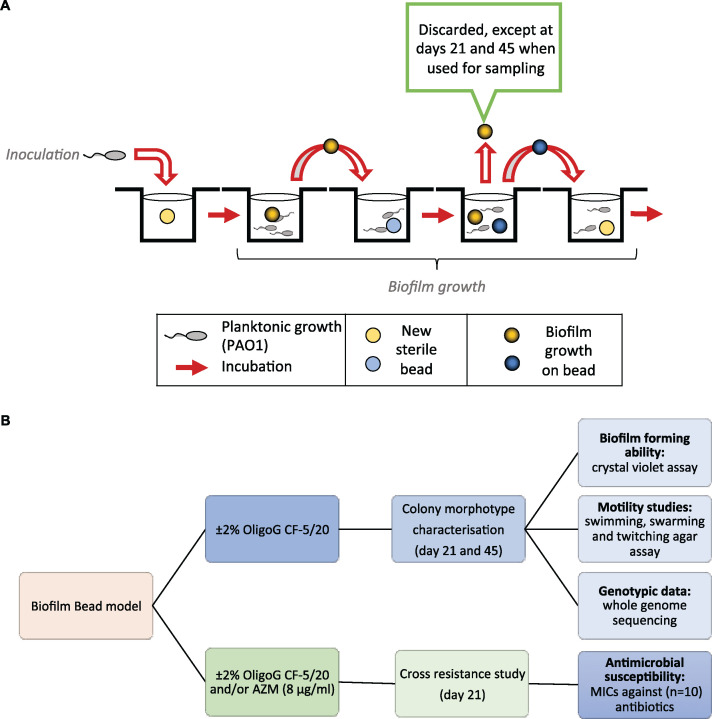
Schematic showing the bead biofilm model experimental strategy. (A) Modeling of prolonged exposure to OligoG CF-5/20 and azithromycin exposure. (B) Corresponding experimental design flowchart (AZM; azithromycin). Biofilms were grown on sterile 7-mm-diameter borosilicate glass beads with yellow and blue beads used for alternate transfer days.

A bead biofilm model was employed with Pseudomonas aeruginosa (PAO1) with or without 2% OligoG CF-5/20 over 45 days (Fig. 1) (see Materials and Methods). Briefly, biofilms were formed on the surface of beads in wells containing growth medium with or without 2% OligoG CF-5/20. On passage days, biofilm-covered beads were transferred into a new well containing fresh medium (± 2% OligoG CF-5/20) alongside a new sterile bead. The bacteria from the mature biofilm community would then colonize the new sterile bead, to repeat the transfer sequence again. The “old” beads were either discarded or used for sampling on transfer days 21 and 45 (Fig. 1A).

**FIG 2:**
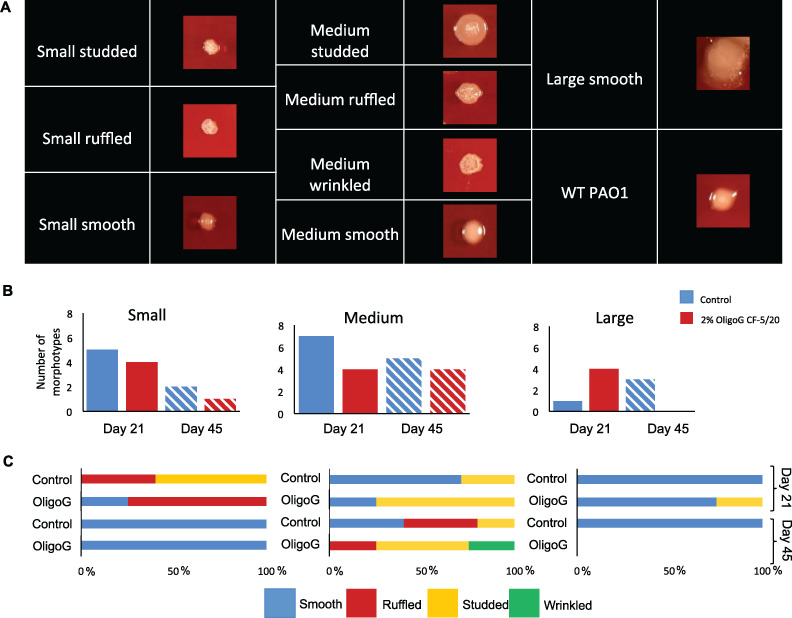
Prolonged exposure of P. aeruginosa results in decreased morphotype diversity. (A) Appearance of representative single-colony morphologies on blood agar plates from control and 2% OligoG plates, shown to scale (small [pinpoint], <1 mm; medium, 1 to 3 mm; large, >3 mm). Comparison of biofilm beads exposed to 0% and 2% OligoG over 21 and 45 days. (B) Categorizing numbers of morphotypes (small, medium, and large colonies) isolated from each growth condition. (C) Subcategorizing surface textures of small, medium, and large colonies (*n* = 4).

As a defined core set of SCV genes has yet to be documented (and with commonality between phenotypic and genotypic changes within different SCV populations not always apparent), for the purposes of this study SCVs were defined as pinpoint colonies formed within 72 h (<1 mm in diameter) (28). Individual bacterial colony morphotypes were characterized according to size ([a] small [pinpoint], <1 mm; [b] medium, 1 to 3 mm; and [c] large, >3 mm) and surface texture (Fig. 2A), as well as opacity, mucoidy, and colony margin (see Table S1 in the supplemental material). Phenotypic characterization of the biofilm-evolved monoculture isolates (identified within the agar plate) revealed that 40 different morphotypes were evident across the study as follows from 4 biological repeats: day 21 control, 13 isolates; day 21 OligoG CF-5/20, 12 isolates; day 45 control, 10 isolates; and day 45 OligoG CF-5/20, 5 isolates (Table S1).

10.1128/mSphere.01216-20.1 TABLE S1 Morphotypes isolated from the bead biofilm model. Categorizing morphotypes isolated from the wells in the presence and absence of 2% OligoG CF-5/20 at days 21 and 45 of bead transfer. Download Table S1, DOCX file, 0.4 MB. Copyright © 2021 Oakley et al. 2021 Oakley et al. This content is distributed under the terms of the Creative Commons Attribution 4.0 International license.

Half of all morphotypes proved to be medium-sized colonies which were less frequently isolated in the OligoG CF-5/20-treated samples at day 21 (Fig. 2B). Only a small proportion were large morphotypes, with none seen by day 45 in the 2% OligoG CF-5/20 samples. A decrease in morphotype diversity was evident in both the control and OligoG CF-5/20-treated group by day 45. SCVs were evident across the study, with fewer morphotypes seen from days 21 to 45 in both control and treated groups.

### Characterization of SCVs.

A total of 12 different small colony variant (SCV) morphotypes were isolated from the samples. One biological repeat had no SCVs in either the control or the OligoG CF-5/20 sample at any time point (Table S1). The remaining three biological repeats demonstrated a reduction in SCV morphotype diversity, with 1 smooth SCV morphotype isolated at day 45.

The surface textures of the SCVs were more variable at day 21 than at day 45 (Fig. 2C), being ruffled and studded in the control samples and smooth and ruffled in the 2% OligoG CF-5/20-treated samples at day 21. These characteristics were lost by day 45, with all clones demonstrating smooth colony morphologies. In contrast, the medium-sized colonies demonstrated both smooth and studded colonies at day 21, which actually became more diverse by day 45 for both control (smooth, ruffled, and studded) and OligoG CF-5/20 (ruffled, studded, and wrinkled) samples. The majority of the large phenotypes isolated over the duration of the study were smooth.

### Prolonged treatment of PAO1 with OligoG CF-5/20 reduces the biofilm-forming ability of small and medium-sized morphotypes.

**FIG 3:**
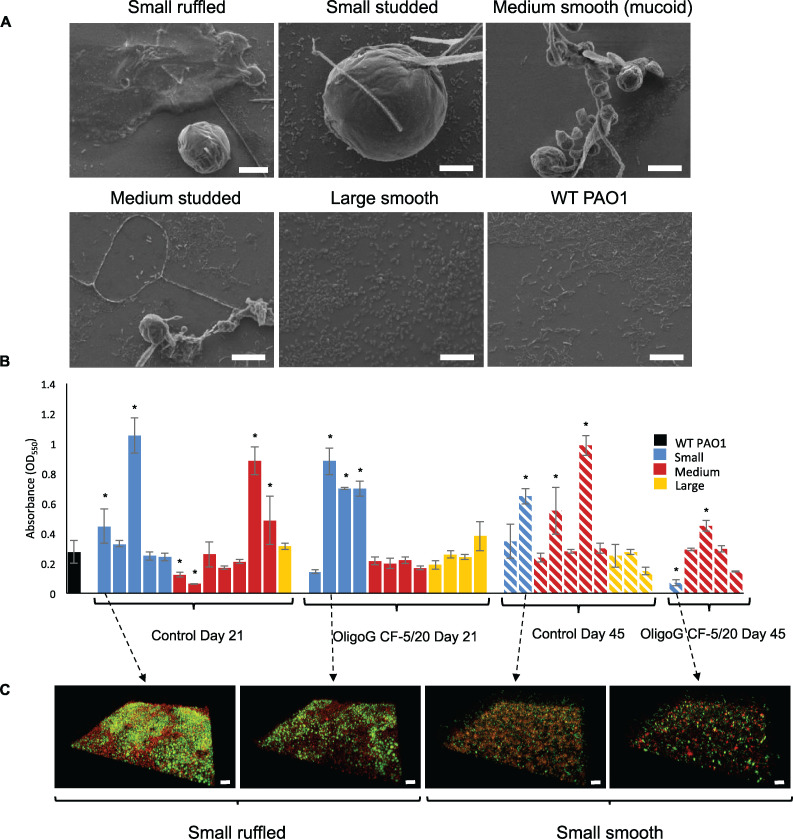
Prolonged exposure to OligoG CF-5/20 alters the biofilm-forming ability of the colony morphotypes. (A) Biofilm-forming ability of selected morphotypes from control samples using scanning electron microscopy (day 21; bar, 10 μm). (B) Crystal violet quantification of biofilm-forming ability of all morphotypes from control and 2% OligoG CF-5/20 biofilm wells (24 h). (C) Confocal laser scanning microscopy imaging of selected SCV biofilms (LIVE/DEAD stained; bar, 20 μm) (*, *P* < 0.05; *n* = 3).

The morphology of biofilms produced by the different morphotypes evolved during the bead biofilm model was investigated using scanning electron microscopy (SEM), crystal violet (CV) biofilm assays, and confocal microscopy (*n* = 3). Untreated control samples were visualized under SEM, according to their subclassification (at 21 days). Small, ruffled, and studded clones demonstrated formation of medium to large biofilm microcolonies, with the former subtype also forming EPS-encased biofilms bound to the glass surface (Fig. 3A). Medium-sized clones formed small clusters of microcolonies, interlinked with branching cells. The smooth mucoidal subtype did not attach strongly to the glass surface, while the studded subtype produced a combination of spherical microcolonies and more flat homogenous biofilms attached to the glass surface. Large clones demonstrated a similar monolayer biofilm to that found in the wild-type (WT) PAO1, although the cells appeared more rounded in shape.

The crystal violet (CV) binding assay (29, 30) highlighted the inherent variability between the biofilm-forming abilities of the different morphotypes (Fig. 3B). The large colonies showed similar values to that of the WT (*P* > 0.05). The medium-sized colonies in the control samples had an increased biofilm-forming ability compared to those found in the OligoG CF-5/20-treated samples at day 21 and day 45. The majority of the treated and untreated SCVs demonstrated an increased biofilm-forming ability compared to the WT. The day 45 SCV isolated from the OligoG CF-5/20-treated sample contrarily exhibited a statistically significant reduction in its biofilm-forming ability (*P* < 0.05, Fig. 3B).

Confocal laser scanning microscopy (CLSM) of 24-h biofilm-grown SCVs (LIVE/DEAD stained) highlighted the variability evident between the biofilm forming abilities of SCVs (Fig. 3C). Biofilms formed from the small smooth morphotypes at day 45 demonstrated a less dense and sparser biofilm in the treated samples than in the untreated control, confirming the findings in the CV assay.

### Altered motility profile of the OligoG CF-5/20-treated morphotypes.

**FIG 4:**
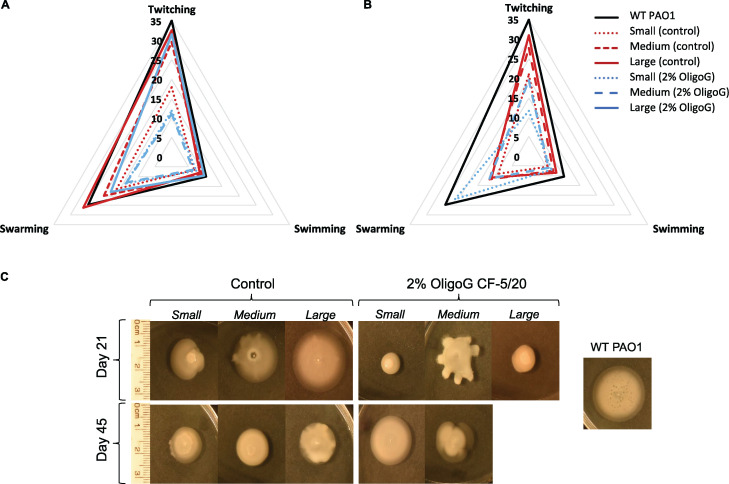
Changes in the motility of control and 2% OligoG CF-5/20 morphotypes. Radar chart demonstrating median twitching, swimming, and swarming motility (millimeters) compared to WT control. (A) At day 21. (B) At day 45. (C) Example of swarming motility plates for small, medium, and large colonies. (No large colonies were isolated from OligoG CF-5/20 cultures at day 45.) *n* = 3.

SCVs and medium colonies isolated at day 21 (Fig. 4A) and day 45 (Fig. 4B), from both control and OligoG CF-5/20-treated samples, were found to have reduced motility (particularly swarming ability) compared to the WT PAO1 control (Fig. 4A and B). These reductions were more noticeable for the day 45 morphotype isolates, where even the large colonies showed a reduction in motility. Swarming motility was largely unchanged for all isolates at both time points, remaining similar to that of the WT control (Fig. 4A and B). Unlike the other isolates, the SCV morphotype in the 2% OligoG CF-5/20-treated samples at day 45 surprisingly demonstrated an increase in swimming motility (Fig. 4C). However, none of these trends reached statistical significance in motility changes between WT PAO1 control and the morphotypes (control and OligoG CF-5/20-treated samples; *P* > 0.05).

### Genetic diversity of PAO1 isolates evolved in OligoG CF-5/20 demonstrates no evidence of adverse selective pressure.

Genome resequencing was performed to determine genetic changes associated with the evolution of PAO1 biofilm populations, with or without exposure to 2% OligoG CF-5/20. Overall, 96 mutations (single nucleotide polymorphisms [SNPs], insertions, deletions, duplications) were identified across 38 bead biofilm-evolved isolates, with two day 21 transfer isolates (C10a and O6a) having no evidence of genomic mutation. Eight mutations were in noncoding regions, while 88 were in coding regions. The 8 mutations in noncoding regions were all identified in control day 45 transfer isolates (C1b, C2b, and C3b) and excluded from the overall “functional” analysis. The 88 muations in coding regions represented 39 unique changes, affecting 21 coding regions (see Table S2 in the supplemental material). Only five mutations were synonymous, 1 was found in the *tssL1* gene in the isolate C3b, and two mutations were found in a gene encoding a hypothetical bacteriophage-associated protein in two isolates, C1b and C2b. Analysis of the distribution of the 88 mutations revealed that there was a significantly higher number of mutations in the day 45 transfer isolates than the day 21 transfer isolates (control, day 21 versus day 45, *P* = 0.02; OligoG CF-5/20, day 21 versus day 45, *P* = 0.02). There was no difference, however, in the numbers of mutations observed between control and OligoG CF-5/20-exposed isolates (control versus OligoG CF-5/20, day 21, *P* = 0.28; day 45, *P* = 0.90).

10.1128/mSphere.01216-20.2 TABLE S2 Mutation detection by whole-genome resequencing of evolved biofilm isolates. Download Table S2, DOCX file, 0.03 MB. Copyright © 2021 Oakley et al. 2021 Oakley et al. This content is distributed under the terms of the Creative Commons Attribution 4.0 International license.

Genes that had acquired mutations in the evolved isolates were clustered according to function (Table 1). The majority of mutations occurred in genes associated with signal transduction (*n* = 32) or transcription (*n* = 39) and genes encoding hypothetical or bacteriophage-associated hypothetical proteins (*n* = 8), with smaller numbers of mutations linked to motility (*n* = 5), secretion (*n* = 1), and translation (*n* = 3). Several genes in the signal transduction and transcription functional categories were found in pathways involved in biofilm formation, chemotaxis, motility, and QS. Apart from the *mvfR* and *mexT* transcriptional regulators and the *wspF* methyltransferase involved in signal transduction, no other genes were found to have mutations in isolates from all four populations. Notably, acquired mutations in motility genes were present only, in both control and OligoG CF-5/20-treated isolates, at day 45.

**TABLE 1:**
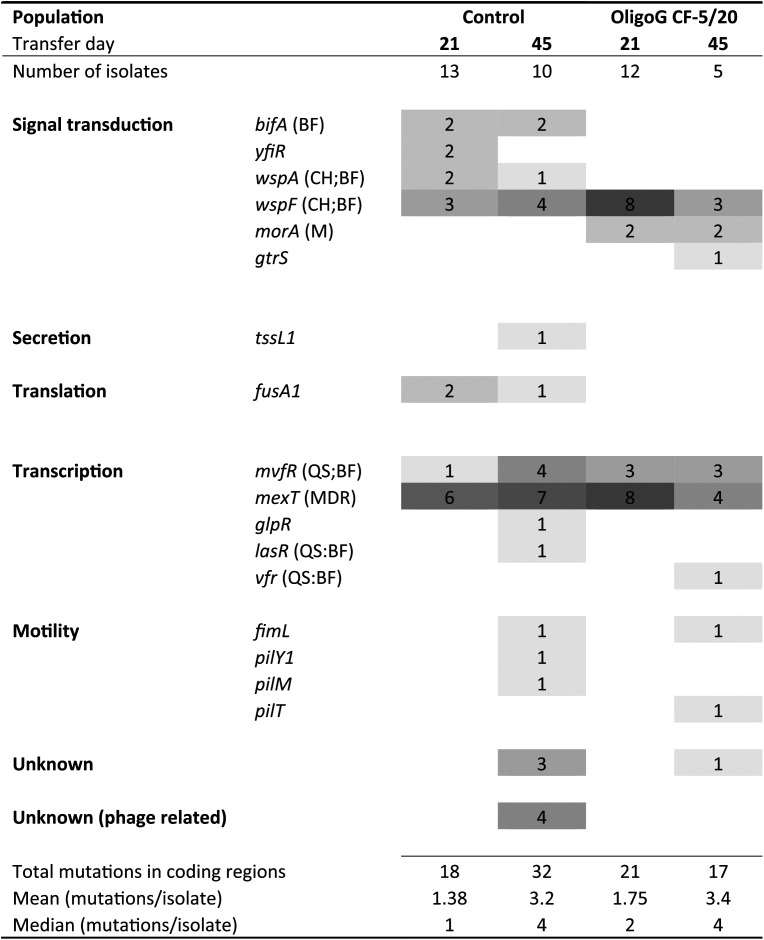
Distribution of mutations in evolved genotypes^*a*^

a The heatmap indicates the numbers of mutations in genes belonging to different functional groups, as per the *Pseudomonas* Genome Database. The color intensity reflects the frequency of mutations in each population (actual values also given inside the boxes). Populations are indicated at the top of the figure: control and OligoG CF-5/20 at transfer days 21 and 45. Links to functional pathways are given in parentheses next to gene identifications: BF, biofilm; CH, chemotaxis; M, motility; QS, quorum sensing; and MDR, multidrug resistance.

### Chronic exposure to OligoG CF-5/20 and azithromycin reduces the acquisition of resistance to other classes of antibiotics.

The macrolide azithromycin (AZM) is often prescribed for prolonged use in patients colonized with chronic P. aeruginosa due to its anti-inflammatory effects (27). In a parallel experiment, the effect of the antibiotic AZM (in the presence and absence of 2% OligoG CF-5/20) was also examined in the bead model over 45 days (Fig. 1B).

The enriched mixed populations (using the day 21 biofilm beads cultured for 24 h in fresh medium) were tested for cross-resistance against a range of antibiotics commonly used in CF or representatives of different classes of antibiotics with different mechanisms of action (*n* = 10). Antibiotics targeting key CF pathogens (including chronic P. aeruginosa) used within the cross-resistance study were comprised of azithromycin (macrolide) (27), ciprofloxacin and levofloxacin (quinolones), colistin (polymyxin E), and aztreonam (monobactam) and tobramycin (aminoglycoside). As cephalosporins, carbapenem, rifamycin, and tetracyclines are also routinely used for CF treatment, ceftazidime, meropenem, rifampin, and oxytetracycline, respectively, were also employed in the cross-resistance studies (31).

Prolonged exposure of PAO1 (grown in the presence and absence of 2% OligoG CF-5/20) demonstrated no change in resistance to azithromycin, both values being 32 μg/ml. However, subculturing at sub-MIC levels of AZM (8 μg/ml) in both the presence and absence of 2% OligoG CF-5/20 resulted in an increase in MIC values from 32 μg/ml to 256 μg/ml at day 21 (Table 2). This resistance to AZM (± OligoG CF-5/20) was retained for up to 6 subsequent transfers (with no antibiotics) following completion of the 45-day experiment.

**TABLE 2:** The effect of OligoG CF-5/20 on the acquisition of resistance to azithromycin (AZM) on the whole bacterial population at day 21^*a*^

Treatment	AZM	CAZ	CIP	Oxy-TET	LEV	COL	ATM	MER	RIF	TOB
AZM	256	512	8	128	8	1	8	2	64	0.5
AZM and 2% OligoG	256	128	4	16	4	0.25	1	0.5	64	1

a Data are MIC values (μg/ml) of cross-resistance to other classes of antibiotics. Antibiotics used: CAZ, ceftazidime; CIP, ciprofloxacin; AZM, azithromycin; Oxy-TET, oxytetracycline; LEV, levofloxacin; COL, colistin; ATM, aztreonam; MER, meropenem; RIF, rifampin; TOB, tobramycin. Decrease in antimicrobial activity (MIC) is indicated by shaded areas.

Samples grown in Mueller-Hinton (MH) broth only or 2% OligoG CF5-/20 demonstrated no change in resistance to other classes of antibiotics (Table S3). Biofilms grown in AZM with 2% OligoG CF-5/20 demonstrated a decrease (up to 3-fold) in resistance (MIC) to antibiotics such as aztreonam and oxytetracycline compared to biofilms growth in AZM alone (Table 2).

10.1128/mSphere.01216-20.3 TABLE S3 OligoG CF-5/20 exposure does not lead to cross-resistance of P. aeruginosa in the bead biofilm model. Whole-population samples at days 21 and 45 tested against a range of antibiotics (CAZ, ceftazidime; CIP, ciprofloxacin; AZM, azithromycin; oxy-TET, oxytetracycline; LEV, levofloxacin; COL, colistin; ATM, aztreonam; MER, meropenem; RIF, rifampin; TOB, tobramycin). Download Table S3, DOCX file, 0.05 MB. Copyright © 2021 Oakley et al. 2021 Oakley et al. This content is distributed under the terms of the Creative Commons Attribution 4.0 International license.

## DISCUSSION

Large-scale evolutionary research has provided extensive evidence on the transition of P. aeruginosa from opportunistic to primary pathogen in the CF lung (13). P. aeruginosa lung infections typically progress clonally following infection, often with a single environmentally acquired genotype (10). Over the course of long-term chronic CF infection, clonal populations undergo considerable phenotypic and genetic adaptation to the lung environment (8, 32) alongside widespread biofilm formation (33). We used the biofilm bead model to mimic this long-term, adaptive process *in vitro*, to assess the phenotypic and genotypic changes in P. aeruginosa biofilm populations following prolonged treatment with the antimicrobial agent OligoG CF-5/20.

*In vitro* short-term experiments (up to 7 days) have shown that biofilm communities undergo extensive diversification (32), but paradoxically, our study showed that this was not evident over the longer term. The demonstration here of decreased morphotype diversity present in our biofilm-bead model at day 45 more closely reflects the findings of *in vivo* studies showing limited diversification of the evolving P. aeruginosa lineage (13). It has been hypothesized that within biofilm communities, bacteria are effectively exposed to lower (sublethal) concentrations of antibiotics (leading to low-level mutation rates) than bacteria growing planktonically (34). Taken together, these findings support the validity of the bead model, as it appears to closely mimic the *in vivo* situation.

SCVs are readily isolated from CF lung-adapted P. aeruginosa populations; having been associated with poor clinical outcome in CF patients (35) and the persistence of infection in animal models (36). Whether they are etiologically involved in the disease process or just preferentially associated remains unclear (11). *In vitro* biofilm studies have previously shown the rapid emergence of SCVs in P. aeruginosa within a 5-day biofilm grown in a drip flow reactor (32). We observed the emergence of SCVs within 21 days. However, cultures containing SCVs had decreased by day 45. SCVs are characterized by their slow growth (37) and hyperdetachment phenotype (32). Mutations such as hyperdetachment can enable bacteria to escape environmental stresses, (e.g., antibiotic treatment and nutrient limitation), enhancing dissemination and colonization of communities (13). Samples subcultured in the presence and absence of OligoG CF-5/20 exhibited fewer SCVs by day 45 than by day 21; however, the only SCV isolated from the OligoG CF-5/20-treated samples at 45 days had a reduced biofilm-forming ability and increased motility. The phenotypic behavior of the SCV formation following growth in OligoG CF-5/20 may have significant clinical advantages for the CF population.

Studies have previously shown that subcultured biofilm populations diversified into smooth/studded (S), ruffled spreader (R), and wrinkly (W) phenotypes (24). While prolonged transfer led to a decrease in the ruffled spreader and wrinkly phenotypes, it has been hypothesized that smooth phenotypes may persist (24), a trend observed here in the surface texture of the SCVs from both control and OligoG CF-5/20-treated biofilm samples. Even without antibiotic selection, biofilm drip flow reactor model studies, using various PAO1 mutants (and clinical CF isolates), produced a variety of colony variants similar to those observed in the reference strain, likely due to spontaneous, *recA*-dependent, recombination events (increasing the competitive advantage of the biofilm-growing cells to resist environmental stress) (32).

Changes in motility phenotype have previously been observed early in biofilm culture (at 5 days), which have been hypothesized to be caused by multiple genetic changes (32). Our finding of reduced motility of all pseudomonal SCVs (compared to the WT PAO1) observed at day 21 has previously been demonstrated (38). However, at 45 days, the single OligoG CF-5/20-treated biofilm SCV isolate retained its ability to swim and had a reduced biofilm-forming ability, indicating that it did not appear to behave like a “typical” SCV.

The spectrum of mutations observed in the biofilm isolates is consistent with previous work on P. aeruginosa. All but five of the observed mutations were nonsynonymous, reflecting that adaptation to the biofilm lifestyle proceeds via higher proportions of nonsynonymous mutations (39, 40). Similar nonsynonymous mutations have been noted *in vivo* in Burkholderia dolosa where adaptive evolution in parallel was also demonstrated, in multiple individuals (41). *In vivo* studies (undertaken over 35 years) have shown that phenotypic changes in the bacterial CF biofilms decrease with time, while genotypic changes were found to be more consistently linear over the same time period (13). Furthermore, *in vivo* CF-evolved phenotypes have shown a discordant relationship between phenotypic characteristics when multiple isolates from the same sample were tested, and even colonies categorized as the same morphotype have been shown to display different antimicrobial susceptibility profiles (42). It is perhaps unsurprising that the separate *in vitro* cultures have evolved divergently, as *in vivo*, P. aeruginosa bacteria inhabiting different lobes of the CF lung have been shown to possess diverse and different phenotypes (43).

Of the 6 signal transduction genes that had acquired mutations, five (*bifA*, *yfiR*, *wspA*, *wspF*, and *morA*) were linked to regulation of intracellular cyclic-di-GMP levels, being closely involved in the transition from motile to sessile lifestyles (44). In particular, both *wspF* and *morA* have been implicated in colony morphology changes (wrinkled colonies and SCVs) and biofilm adaptation in P. aeruginosa (45). In isolates from the day 45 transfer populations, genes directly related to motility including type IV pili (*pilY1*, *pilM*, and *pilT*) were mutated, as has been observed previously in P. aeruginosa PAO1 biofilm models (34, 40). Overall, the majority of mutated genes were linked to pathways involved in biofilm formation, chemotaxis, motility, and QS pathways, corroborating the results of other studies and the pathoadaptive mutations frequently observed in P. aeruginosa within the CF lung (12, 46).

Experimental evolutionary studies have investigated genomic mutation in P. aeruginosa following exposure to antibiotics such as ciprofloxacin and demonstrated a significant increase in the number of mutations in both planktonic and biofilm populations (34), as well as in populations modeled in synthetic CF sputum medium (45). These studies found mutations in genes specifically linked to ciprofloxacin exposure (Mex efflux systems, DNA gyrases) and genes associated with adaptation to a CF-like environment (QS, motility, cyclic-di-GMP signaling). While we observed mutations in the *mexT* gene, a multidrug efflux transcriptional regulator (47), these occurred across all populations. Moreover, while a significant increase in the number of mutations was observed between day 21 and day 45 transfer populations, these were similar in control and OligoG CF-5/20-exposed isolates. Furthermore, there were no mutated genes in functional gene categories exclusive to OligoG CF-5/20-exposed isolates, the same categories being affected in both control and OligoG CF-5/20-exposed populations. From this prolonged exposure experiment, we conclude that, unlike the antibiotics tested, OligoG CF-5/20 did not drive mutations in specific genes, during the adaption of P. aeruginosa PAO1 to biofilm growth.

Clearly, these experiments lack the highly selective and heterogeneous local environmental stressors (e.g., oxidative and osmotic stress) found in the CF lung (48), which are important in bacterial evolution (10). However, clinical studies have highlighted sampling variability and microbial diversity due to CF lung compartmentalization, making analysis of clinical samples difficult (49). Clark et al. also showed that the abundance of individual morphotypes fluctuated with time, no single morphotype predominating in the 12 samples collected over a 12-month period and considerable phenotypic variability being observed in isolates from individual sputum samples (42). The advantage of laboratory models such as the bead model used here is therefore apparent, being able to produce reproducible biofilms under controlled conditions.

The effect of prolonged exposure to commonly utilized antibiotics at sub-MIC levels positively selects for resistant bacteria (50). Clinical studies have shown rapid shifts in antimicrobial susceptibility profiles within short periods of time (as short as 7 days) following antibiotic administration (32). A clear trend of increased antibiotic resistance over time is also evident within the CF lung microbiome, although considerable intra- and intersample diversity in susceptibility to specific antibiotics is evident (42). Resistance to antibiotics such as aztreonam and ceftazidime was found to be moderately correlated, with discontinuation of aztreonam therapy reducing susceptibility to ceftazidime, and also to ciprofloxacin in individual isolates (42). A parallel study of laboratory adaptation of Escherichia coli to various antibiotics over 90 days demonstrated that acquisition of resistance to a single antibiotic can alter susceptibility to a range of other antibiotics (51).

The mutations incurred can also compensate for fitness cost associated with the acquisition of resistance. Mutants formed in the presence of azithromycin within this study demonstrated altered susceptibility to other classes of antibiotics. This highlights a possible alternative mode of action for mutations arising in the presence of OligoG CF-5/20, where P. aeruginosa was rendered more susceptible to antibiotics such as aztreonam and oxytetracycline. We have previously observed antibiotic potentiation with OligoG CF-5/20 in planktonic culture (16, 17). This study confirmed that the presence of OligoG CF-5/20 did not alter the acquisition of resistance to azithromycin within these biofilms when assessed at day 21. These findings suggest that bacteria growing *in vitro* in the presence of OligoG CF-5/20 have fewer colonies with MDR-associated phenotypes and improved antibiotic susceptibilities, which may afford significant clinical benefits in patients treated with this novel antibiofilm therapy.

## MATERIALS AND METHODS

### Bacterial strain and culture conditions.

Pseudomonas aeruginosa (PAO1, ATCC 15692) was used throughout the study. Overnight cultures (*n* = 4) were prepared in tryptone soy broth (TSB; LabM) using an inoculum from bacteria freshly cultured on blood agar plates (BA; Blood agar base no. 2; LabM), and all experiments were performed in Mueller-Hinton (MH) broth (LabM).

### Alginate oligosaccharide (OligoG CF-5/20).

OligoG CF-5/20 was produced from the stem of the brown seaweed Laminaria hyperborea and provided by AlgiPharma AS (Sandvika, Norway), as previously described (17). OligoG CF-5/20 has a high guluronate content (>85%) and a mean degree of polymerization (DPn) of 16.

### MIC assay.

MIC assays were performed using a broth microdilution method in MH broth as previously described (17) in accordance with standard guidelines (52).

### Bead biofilm evolution model.

The bead model used here was adapted from a previous study (24) (Fig. 1A). Biofilms were grown on sterile borosilicate glass beads (7-mm diameter; with yellow and blue beads used for alternate transfer days) which were placed in each of well of a 24-well microtiter plate containing 1 ml MH broth plus or minus 2% OligoG CF-5/20 (*n* = 4 for each condition/control). Plates were incubated continuously at 37°C (20 rpm) for the duration of the experiment, On passage days, the beads were transferred into the corresponding wells of a fresh 24-well plate 3 times/week, which contained a single new sterile bead (in each well), with the old bead being discarded. On days 21 and 45, however, the old bead was removed from each well and used to inoculate 10 ml sterile TSB for overnight growth at 37°C.

At time zero, one bead was placed in each well in an inoculum of WT PAO1 of 5 × 10^4^ CFU/ml. However, assuming that planktonic cells decline over time (by up to 33%) (24) due to an increase in the number of cells attaching to the bead, the number of generations was estimated at 16× log_2_(dilution), giving an estimate for generations over the course of the experiment between 240 and 250. Each plate contained a sterile MH broth control. Purity plates were performed weekly on each test well on BA plates. At days 21 and 45, purified morphotypes were frozen at −80°C on microbank beads to be used for subsequent morpho- and genotyping experiments.

### Phenotypic characterization of colony morphotypes.

The “old” bead was removed, vortexed in fresh TSB, and grown overnight before samples were plated out onto blood agar and incubated for 24 to 72 h before purifying individual colonies on fresh BA plates and freezing them for later use. The colony morphotypes isolated (MH ± 2% OligoG CF-5/20) were reviewed independently by two researchers at both day 21 and day 45 to ensure consistency of morphological identifications.

### Scanning electron microscopy (SEM).

A selection of different morphotypes in relation to size and surface textures was made from the control sample at day 21 (small ruffled, small studded, medium smooth [mucoid], medium studded, and large smooth) and a WT PAO1 control. Overnight cultures of individual morphotypes were adjusted to 10^6^ CFU/ml in MH broth and grown for 24 h in a 12-well plate (Greiner Bio-One) on glass slides (37°C; 20 rpm). Following incubation, the supernatant was removed, and biofilms were fixed with 2.5% (vol/vol) glutaraldehyde prior to being washed (4 times) with distilled water (dH_2_O) and freeze-dried. Samples were gold coated and imaged using a Tescan Vega conventional scanning electron microscope (SEM; 6 kV).

### Biofilm formation crystal violet assay.

All morphotypes from day 21 and day 45 were tested for biofilm-forming ability in 96-well polystyrene plates and compared to the WT PAO1 control. Biofilms were grown in a flat-bottomed 96-well plate (5 × 10^5^ CFU/ml in MH broth) for 24 h statically (*n* = 3 biological, *n* = 5 technical repeats) and quantified using an adapted crystal violet (CV) methodology (29, 30). The plates were gently washed (twice) in dH_2_O (for 10 s) to remove planktonically growing cells prior to adding 0.1% CV (125 μl) into each well to stain the attached cells. Following a 15-min incubation at room temperature, plates were rinsed in dH_2_O (4 times) and allowed to dry for ≥1 h, prior to solubilizing the dye in 95% ethanol (200 μl for 30 min). A total of 125 μl per well was removed to a fresh 96-well plate, and the absorbance (optical density at 550 nm [OD_550_]) was measured with a spectrophotometer.

### Confocal laser scanning microscopy (CLSM) imaging of SCVs.

Individual isolates representing smooth and ruffled small colonies from the SCVs were chosen from biofilm monocultures obtained on day 21 and day 45 (control and 2% OligoG CF-5/20 plates). When multiple samples were available, clones with the best biofilm production were selected. Overnight cultures were prepared in TSB from the freezer stock (37°C; 120 rpm). CLSM was performed on biofilms grown in Grenier 96-well glass-bottomed plates in MH broth using a starting inoculum of 10^6^ CFU/ml (37°C; 24 h). Supernatant was removed before staining the cells with 6% (vol/vol) LIVE/DEAD (BacLight bacterial viability kit; Invitrogen) stain in phosphate-buffered saline (PBS) for 10 min, prior to imaging with a Leica SP5 confocal microscope with ×63 magnification under oil.

### Motility assays.

All morphotypes from day 21 and day 45 were tested for swimming, swarming, and twitching ability compared to the WT PAO1 control. Agar plate-based assays were prepared, and assay mixtures were poured and used immediately once set, as previously described (53). Overnight cultures were prepared from freezer stocks in TSB, and cells were point inoculated with a sterile toothpick onto the surface of the agar prior to being incubated for 48 h at 25°C (swimming) or 30°C (swarming) and 72 h at 30°C (twitching). Motility was determined as the widest diameter of bacterial migration (millimeters).

### Genotypic characterization of colony morphotypes.

Whole-genome sequencing was performed following genomic DNA extraction from wild-type P. aeruginosa PAO1 and evolved PAO1 isolates using the Maxwell instrument and Maxwell 16 tissue DNA purification kits (Promega) according to the manufacturer’s instructions. Briefly, 3 ml of fresh overnight culture was pelleted by centrifugation, and the pellet was resuspended in 300 μl 4 M UltraPure guanidine isothiocyanate (ThermoFisher Scientific) and added directly into the DNA purification kits. Eluted DNA was stored at −20°C. Whole-genome sequencing was performed at the Cardiff School of Biosciences Genomics Research Hub. DNA was prepared for sequencing using the NEBNext Ultra II DNA library prep kit for Illumina and NEBNext multiplex oligonucleotides for Illumina (New England BioLabs Inc.). Sequencing was carried out on an Illumina NextSeq500 using a NextSeq 500/550 Mid Output v2 kit (300 cycles), giving, on average, 135-bp paired-end reads. Approximately 2.9 million reads (range: 2.5 to 3.4 million) were yielded per sample, corresponding to an average coverage depth of approximately 125× (range: 106 to 142×).

### Bioinformatic analysis of whole-genome sequencing data.

Bioinformatic analysis was carried out on a virtual machine, hosted by the Cloud Infrastructure for Microbial Bioinformatics (CLIMB) consortium (54). Quality control and Illumina adapter trimming of the raw sequencing reads were performed using FastQC v0.11.5 (55) and Trim Galore! v0.4.3 (56) for paired-end reads. Genome assembly for the PAO1 WT was achieved using Unicycler v0.4.7 (57) with SPAdes v3.11.0 (58) and the option for short-read assembly. Assembly quality was visualized with Bandage assembly graphs (59) and with QUAST v4.6.3 (60) to determine that the PAO1 wild type shared >98.8% genomic DNA with the P. aeruginosa PAO1 ATCC 15692 sequence (GenBank accession number GCA_001729505.1). Contig ordering to the P. aeruginosa PAO1 ATCC 15692 sequence was performed using ABACAS v1.3.1 (61). The resulting draft genome sequence was annotated with Prokka v1.12 (62).

Polymorphic sites in the evolved PAO1 isolates were identified using Snippy v3.2 (63) with the draft genome sequence of the wild-type PAO1 as the reference. For variant calling, the default parameters of minimum base quality of 20, minimum read coverage of 10×, and 90% read concordance at each locus were used. Only variants in the annotated coding regions were included in the analysis. Variants identified in the wild-type PAO1 sequence reads were subtracted from all other evolved PAO1 isolates. Correct annotation of coding sequences containing variants was confirmed using the BLASTN search tool of the *Pseudomonas* Genome Database (64) against the P. aeruginosa PAO1 reference sequence. Functional information for coding sequences was derived from the *Pseudomonas* Genome Database.

### Acquisition of resistance in the presence of azithromycin.

The rate of acquisition of resistance to azithromycin (AZM) at a sublethal concentration 2-fold lower than the MIC level (AZM; 8 μg/ml) was conducted in the presence and absence of 2% OligoG CF-5/20 using the biofilm bead model (Fig. 1A). Glass beads were placed in 1 ml AZM (8 μg/ml) with or without 2% OligoG CF-5/20 in 24-well plates prior to inoculation with PAO1 cultures (5 × 10^4^ CFU/ml; *n* = 4) and incubated (80 rpm; 37°C). Beads were transferred into the corresponding wells in a fresh plate 3 times/week (Fig. 1A) for 45 days. Purity plates were performed weekly on each test well on blood agar (BA) plates. At days 21 and 45, samples were taken from all wells and frozen at −80°C on microbank beads to be used for cross-resistance studies.

### Cross-resistance study.

The enriched mixed populations grown in the presence and absence of 2% OligoG CF-5/20 (± AZM) were tested for cross-resistance against a range of antibiotics commonly used (ceftazidime [CAZ], ciprofloxacin [CIP], azithromycin [AZM], oxytetracycline [oxy-TET], levofloxacin [LEV], colistin [COL], aztreonam [ATM], meropenem [MER], rifampin [RIF], tobramycin [TOB]). The day 21 biofilm bead cultures were vortexed in fresh medium and grown overnight (37°C; shaken). Cross-resistance was tested using an MIC assay (broth microdilution method) as previously described (17). These were conducted for the 4 biological repeats.

### Loss of resistance.

Following day 45 of transfer for the AZM study, the stability of AZM resistance was analyzed by transferring beads into MH broth or MH broth with 2% OligoG CF-5/20 for a further six transfers. Biofilm growths from each transfer were subcultured onto LB plates with or without AZM (32 μg/ml), and resistance to AZM was recorded if there was growth on the plates after 24 h.

### Statistical analysis.

The significance of the crystal violet biofilm assay data was assessed using one-way analysis of variance (ANOVA) followed by Dunnett’s multiple-comparison *post hoc* test using Graph Pad Prism 8. The motility assay data were analyzed using a nonparametric Kruskal-Wallis test performed on IBM SPSS followed by a *post hoc* adjustment by the Bonferroni correction. Statistical analyses for the genotypic characterization were performed using R statistical software (65) to determine differences between the numbers of mutations in coding regions observed in each population (Control 21 days, Control 45 days, OligoG CF-5/20-exposed 21 days and OligoG CF-5/20-exposed 45 days). As the numbers of mutations in all populations were found to be nonnormally distributed (Shapiro-Wilk test) with equal variances (Levene’s test), a nonparametric Kruskal-Wallis test with *post hoc* pairwise comparisons using the Wilcoxon rank sum test and Benjamini-Hochberg adjustment was used to determine differences between the population medians. Differences were considered significant for *P* ≤ 0.05.

### Data availability.

Data generated and analyzed during this study are included in this published article and its supplemental material. Sequence data supporting the genomic analysis have been deposited in the European Nucleotide Archive with the accession code PRJEB36146 (ERP119298). Additional details are available upon reasonable request.
