# Supplementary material for: Phenotypic and Genotypic Adaptations in Pseudomonas aeruginosa Biofilms following Long-Term Exposure to an Alginate Oligomer Therapy
Source: mSphere. 2021 Jan 20;6(1):e01216-20. doi: 10.1128/mSphere.01216-20 (PMC7845618; doi:10.1128/mSphere.01216-20)
Supplement: TABLE S1 [file mSphere.01216-20-st001.docx]

**SUPPLEMENTARY TABLE 1**


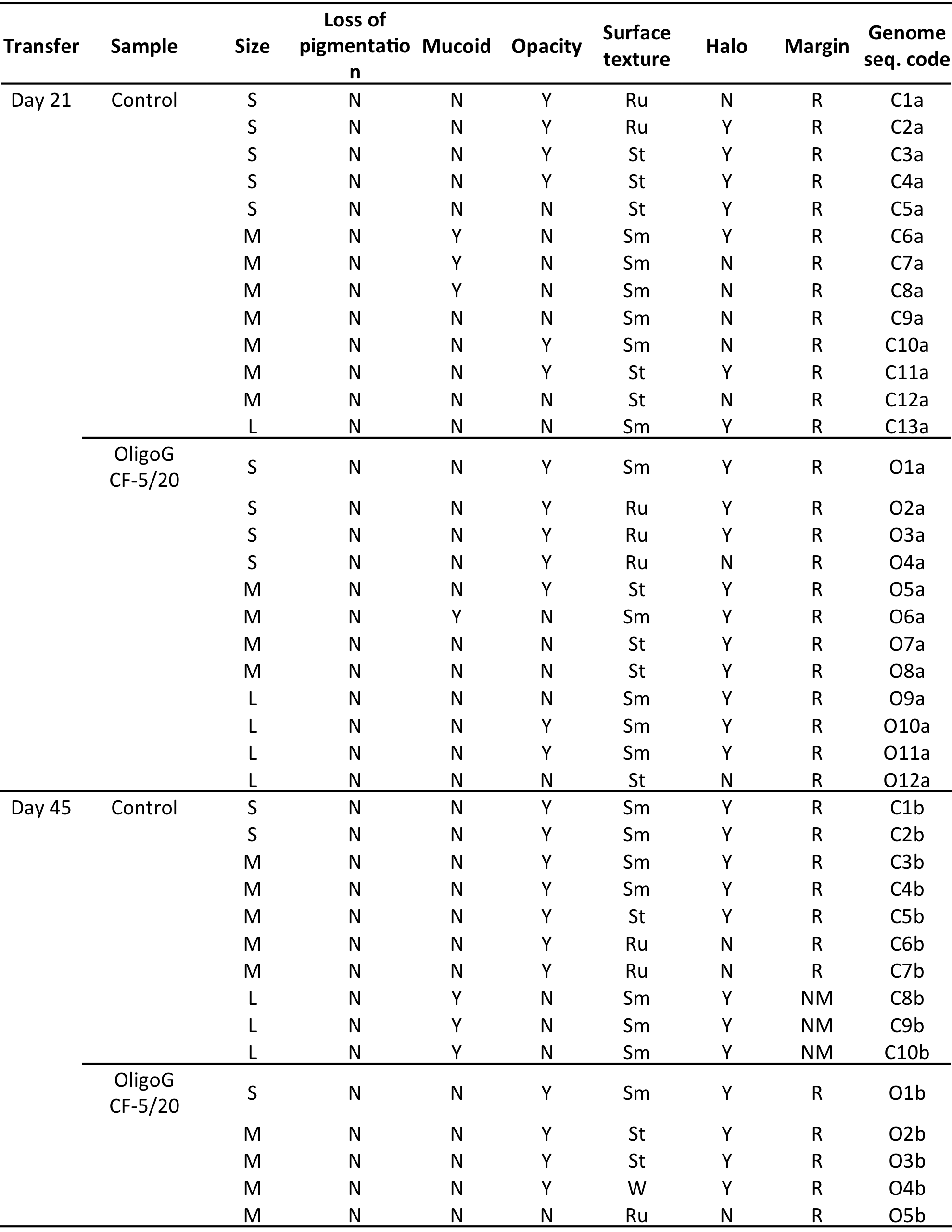


**Key:** S; Small, M; Medium, L; Large, Y; Yes, N; No, R; Round margin, NM; No margin, Ru; Ruffled, St; Studded, Sm; Smooth, W; Wrinkled.
