# Supplementary material for: Phenotypic and Genotypic Adaptations in Pseudomonas aeruginosa Biofilms following Long-Term Exposure to an Alginate Oligomer Therapy
Source: mSphere. 2021 Jan 20;6(1):e01216-20. doi: 10.1128/mSphere.01216-20 (PMC7845618; doi:10.1128/mSphere.01216-20)
Supplement: TABLE S2 [file mSphere.01216-20-st002.docx]

**SUPPLEMENTARY TABLE 2.**

| **Gene with mutation** | **Contig in reference** | **Locus in reference** | **Mutational effect** | **Mutation class** | **Evolved isolate (condition)** | **PGD functional classification*** | **Pathways*** |
| --- | --- | --- | --- | --- | --- | --- | --- |
| **Signal transduction** | | | | | | | |
| *bifA* cyclic-Di-GMP phosphodiesterase | PAO1_108 | PAO1_04439 | missense_variant c.1796A>T p.Lys599Met | Non-synonymous; missense | C6a, C9a,  C3b, C5b | Cell wall/LPS/capsule; Motility and attachment | Biofilm formation |
| *yfiR* protein | PAO1_93 | PAO1_03876 | missense_variant c.404T>A p.Val135Glu | Non-synonymous; missense | C12a | Cell wall/LPS/capsule |  |
|  |  |  | missense_variant c.281T>A p.Val94Glu | Non-synonymous; missense | C11a |  |  |
| *wspA* probable chemotaxis inducer | PAO1_30 | PAO1_01217 | missense_variant c.992C>T p.Ser331Leu | Non-synonymous; missense | C1a, C2a, C2b | Motility and attachment; Adaptation, protection; chemotaxis | Two-component system; Chemotactic transducer (MCP); Chemosensory; Biofilm formation |
| *wspF* probable methylesterase | PAO1_30 | PAO1_01222 | frameshift_variant c.688delA p.Ile230fs | Deletion | C3a, C4a, C6b, C4b, O3a | Chemotaxis; transcriptional regulators; motility and attachment | Two-component system; Chemotaxis; Chemosensory; Biofilm formation |
|  |  |  | frameshift_variant c.880_911delACCATCGCCCAGGACCAGGCCAGTTGCGCAGT p.Thr294fs | Deletion | C5a |  |  |
|  |  |  | frameshift_variant c.445delG p.Ala149fs | Deletion | C1b, C7b |  |  |
|  |  |  | missense_variant c.61G>C p.Ala21Pro | Non-synonymous; missense | O5a, O12a, O7a, O2b |  |  |
|  |  |  | missense_variant c.842C>A p.Ala281Asp | Non-synonymous; missense | O8a |  |  |
|  |  |  | stop_gained c.658C>T p.Gln220* | Non-synonymous; stop | O1a |  |  |
|  |  |  | conservative_inframe_deletion c.37_63delGTCGAGGCGCTGCGCCGCGCGCTGGCC p.Val13_Ala21del | Deletion | O2a |  |  |
|  |  |  | missense_variant c.821C>T p.Thr274Ile | Non-synonymous; missense | O4a |  |  |
|  |  |  | stop_gained c.418C>T p.Gln140* | Non-synonymous; stop | O3b, O1b |  |  |
| Motility regulator *morA* | PAO1_108 | PAO1_04681 | missense_variant c.3464T>A p.Leu1155Gln | Non-synonymous; missense | O11a, O10a, O4b, O5b | Membrane proteins |  |
| Glucose transport sensor *gtrS* | PAO1_36 | PAO1_01743 | missense_variant c.1014C>G p.His338Gln | Non-synonymous; missense | O5b | Two-component regulatory systems | Two-component system |
| Secretion |  |  |  |  |  |  |  |
| *tssL1* membrane protein | PAO1_11 | PAO1_00042 | synonymous_variant c.1137G>A p.Pro379Pro | Synonymous | C3b | Hypothetical, unclassified, unknown; Protein secretion/export apparatus | HCP secretion island (HIS-I) type VI secretion system; Bacterial secretion system |
| **Translation** | | | | | | | |
| Elongation factor G *fusA1* | PAO1_21 | PAO1_00659 | missense_variant c.953C>T p.Ser318Leu | Non-synonymous; missense | C3a | Translation, post-translational modification, degradation |  |
|  | PAO1_21 | PAO1_00659 | missense_variant c.1546G>A p.Gly516Ser | Non-synonymous; missense | C8a, C10b |  |  |
| **Transcription** | | | | | | | |
| Transcriptional regulator *mvfR* | PAO1_93 | PAO1_04000 | conservative_inframe_deletion c.109_120delTCGGCGGTCAGC p.Ser37_Ser40del | Deletion | O11a, O10a, O4b, O5b | Transcriptional regulators; Biosynthesis of cofactors, prosthetic groups and carriers | Quorum sensing; biofilm formation |
|  |  |  | frameshift_variant c.782_785dupGCGG p.Ile263fs | Duplication | C3b, C5b |  |  |
|  |  |  | missense_variant c.101C>T p.Ala34Val | Non-synonymous; missense | O9a |  |  |
|  |  |  | missense_variant c.112G>A p.Ala38Thr | Non-synonymous; missense | O1b |  |  |
|  |  |  | missense_variant c.440T>C p.Ile147Thr | Non-synonymous; missense | C4a, C4b |  |  |
|  |  |  | missense_variant c.527A>C p.His176Pro | Non-synonymous; missense | C6b |  |  |
| Transcriptional regulator *mexT* | PAO1_41 | PAO1_02477 | conservative_inframe_insertion c.389_390insCCT p.Val130_Leu131insLeu | Insertion | C12a, C6a, C2a, C13a, C7a, C5a, C5b, C2b, C9b, C6b, C4b, C1b, C7b, O5a, O7a, O8a, O1a, O2a, O11a, O10a, O4a, O2b, O3b, O1b, O4b | Transcriptional regulators |  |
| Glycerol-3-phosphate regulon repressor *glpR* | PAO1_30 | PAO1_01342 | missense_variant c.169G>A p.Ala57Thr | Non-synonymous; missense | C8b | Transcriptional regulators |  |
| Transcriptional regulator *lasR* | PAO1_93 | PAO1_03564 | missense_variant c.628T>C p.Phe210Leu | Non-synonymous; missense | C6b | Transcriptional regulators; Adaptation, protection | Quorum sensing; biofilm formation |
| Transcriptional regulator *vfr* | PAO1_20 | PAO1_00624 | frameshift_variant c.594dupG p.Leu200fs | Duplication | O5b | Transcriptional regulators | Two-component system; Quorum sensing; Biofilm formation |
| **Motility** | | | | | | | |
| *fimL* protein | PAO1_88 | PAO1_03161 | stop_gained c.1528C>T p.Gln510* | Non-synonymous; stop | O4b | Motility and attachment |  |
|  |  |  | conservative_inframe_insertion c.1340_1341insCCTGGC p.Gly447_Leu448insLeuAla | Insertion | C5b |  |  |
| Type 4 fimbrial biogenesis protein *piLY1* | PAO1_108 | PAO1_04632 | stop_gained c.2993C>A p.Ser998* | Non-synonymous; stop | C6b | Motility and attachment | Pilin biosynthesis |
| Type 4 fimbrial biogenesis protein *pilM* | PAO1_122 | PAO1_05151 | frameshift_variant c.670delG p.Gly224fs | Deletion | C4b | Motility and attachment | Pilin biosynthesis |
| Twitching motility protein *pilT* | PAO1_19 | PAO1_00365 | conservative_inframe_deletion c.970_984delGTCGCCAAGGGCCTG p.Val324_Leu328del | Deletion | O1b | Cell wall/LPS/capsule; Motility and attachment | Pilin biosynthesis |
| **Unknown** | | | | | | | |
| Hypothetical protein | PAO1_79 | PAO1_02747 | conservative_inframe_deletion c.868_888delTTTGAGACTGCTATTTCCCAG p.Phe290_Gln296del | Deletion | O3b |  |  |
|  | PAO1_88 | PAO1_03081 | missense_variant c.523T>C p.Phe175Leu | Non-synonymous; missense | C9b |  |  |
|  | PAO1_120 | PAO1_04769 | frameshift_variant c.281dupC p.Leu95fs | Duplication | C2b |  |  |
|  |  |  | frameshift_variant c.716delC p.Pro239fs | Deletion | C1b |  |  |
| **Unknown (phage related)** | | | | | | | |
| Hypothetical protein from bacteriophage Pf1 | PAO1_100 | PAO1_04289 | synonymous_variant c.246G>T p.Gly82Gly | Synonymous | C2b, C1b |  |  |
|  |  |  | synonymous_variant c.198T>C p.Ser66Ser | Synonymous | C2b, C1b |  |  |

**Footnotes;** PDG, Pseudomonas Genome database; * Functional classifications and pathways according to the Pseudomonas Genome Database
