# Supplementary material for: Phenotypic and Genotypic Adaptations in Pseudomonas aeruginosa Biofilms following Long-Term Exposure to an Alginate Oligomer Therapy
Source: mSphere. 2021 Jan 20;6(1):e01216-20. doi: 10.1128/mSphere.01216-20 (PMC7845618; doi:10.1128/mSphere.01216-20)
Supplement: TABLE S3 [file mSphere.01216-20-st003.docx]

**SUPPLEMENTARY TABLE 3**

| **MIC ug/ml** | **Transfer (day)** | **CEZ** | **CIP** | **AZM** | **Oxy-TET** | **LEV** | **COL** | **ATM** | **MER** | **RIF** | **TOB** |
| --- | --- | --- | --- | --- | --- | --- | --- | --- | --- | --- | --- |
| **Control** | 0 | 16 | 0.125 | 32 | 4 | 0.25 | 1 | 4 | 0.5 | 32 | 1 |
|  | 21 | 16 | 0.0625 | 32 | 8 | 0.5 | 0.5 | 4 | 1 | 64 | 1 |
|  | 45 | 32 | 0.5 | 64 | 8 | 0.5 | 0.5 | 4 | 2 | 32 | 0.5 |
| **2% OligoG CF-5/20** | 0 | 16 | 0.0625 | 32 | 4 | 0.25 | 1 | 4 | 0.5 | 32 | 1 |
|  | 21 | 32 | 0.125 | 64 | 8 | 0.5 | 1 | 4 | 1 | 64 | 1 |
|  | 45 | 16 | 0.125 | 32 | 8 | 0.5 | 0.25 | 4 | 2 | 32 | 0.25 |
